# A Novel 12-Lead Electrocardiographic System for Home Use: Development and Usability Testing

**DOI:** 10.2196/10126

**Published:** 2018-07-30

**Authors:** Annemarijn SM Steijlen, Kaspar MB Jansen, Armagan Albayrak, Derk O Verschure, Diederik F Van Wijk

**Affiliations:** ^1^ Design Engineering Faculty of Industrial Design Engineering Delft University of Technology Delft Netherlands; ^2^ Applied Ergonomics and Design Faculty of Industrial Design Engineering Delft University of Technology Delft Netherlands; ^3^ Department of Cardiology Zaans Medisch Centrum Zaandam Netherlands; ^4^ Department of Cardiology Onze Lieve Vrouwe Gasthuis Amsterdam Netherlands

**Keywords:** 12-lead ECG system, electrocardiography, home use, handheld, user-centered design

## Abstract

**Background:**

Cardiovascular diseases (CVD) are the leading cause of morbidity and mortality worldwide. Early diagnosis is of pivotal importance for patients with cardiac arrhythmias and ischemia to minimize the consequences like strokes and myocardial infarctions. The chance of capturing signals of arrhythmias or ischemia is substantially high when a 12-lead electrocardiogram (ECG) can be recorded at the moment when a patient experiences the symptoms. However, until now, available diagnostic systems (Holter monitors and other wearable ECG sensors) have not enabled patients to record a reliable 12-lead ECG at home.

**Objective:**

The objective of this project was to develop a user-friendly system that enables persons with cardiac complaints to record a reliable 12-lead ECG at home to improve the diagnostic process and, consequently, reduce the time between the onset of symptoms and adequate treatment.

**Methods:**

Using an iterative design approach, *ECGraph* was developed. The system consists of an ECG measurement system and a mobile app, which were developed with the help of several concept tests. To evaluate the design, a prototype of the final design was built and a final technical performance test and usability test were executed.

**Results:**

The ECG measurement system consists of a belt and 4 limb straps. Ten wet Ag/AgCl electrodes are placed in the belt to optimize skin-electrode contact. The product is controlled via an app on the mobile phone of the user. Once a person experiences symptoms, he or she can put on the belt and record ECGs within a few minutes. Short instructions, supported by visualizations, offer guidance during use. ECGs are sent wirelessly to the caregiver, and the designated expert can quickly interpret the results. Usability tests with the final prototype (n=6) showed that the participants were able to put on the product within 8 minutes during first-time use. However, we expect that the placement of the product can be executed faster when the user becomes more familiar with the product. Areas of improvement focus mainly on confidence during product use. In the technical performance test, a 12-lead ECG was made and reproduced 6 times.

**Conclusions:**

We developed a new 12-lead ECG system for home use. The product is expected to be more user-friendly than current hospital ECG systems and is designed to record more reliable data than current ECG systems for home use, which makes it suitable for expert interpretation. The system has great potential to be incorporated into an outpatient practice, so that arrhythmias and ischemia can be diagnosed and treated as early as possible.

## Introduction

### Background

According to the World Health Organization, cardiovascular diseases (CVD) were the leading cause of morbidity and mortality worldwide in 2015 [1]. An estimated 17.7 million people died from CVDs in 2015, representing 32% of all global deaths. Around 7 million people in this group died of ischemic heart disease. Cardiac ischemia refers to decreased blood flow to the heart muscle, resulting in a shortage of oxygen. This can cause damage to or death of a part of the heart muscle known as a myocardial infarction.

Acute ischemic heart disease and myocardial infarction are diagnosed, among others, with the help of a pivotal test: the registration of an electrocardiogram (ECG). The test is performed when a patient presents with acute chest pain, after which the treatment and prognosis can be determined immediately.

**Figure 1:**
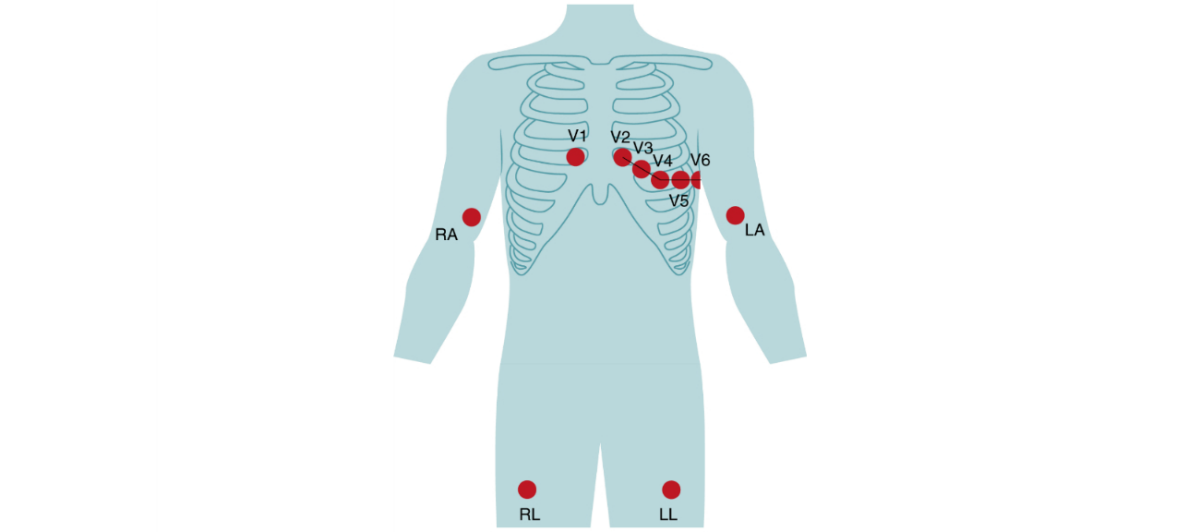
Placement of the electrodes of the standard 12-lead electrocardiographic system; LA: left arm; LL: left leg; RA: right arm; RL: right leg; V1 to V6: the 6 precordial electrodes.

A standard ECG measures the depolarization of cardiac muscle tissue [2] and consists of 12 leads, recorded from 10 electrodes that are placed on the skin (Figure 1). The leads gather information from different sides of the heart. To obtain a complete and reliable overview of heart activity, all 10 electrodes need to be placed at the prescribed locations shown in Figure 1 [3].

It is estimated that 1.5 million to 3 million ECGs are performed worldwide every day, making it one of the most commonly used cardiovascular diagnostic procedures and a fundamental tool in clinical practice [4,5]. For patients with ischemia, early diagnosis is of great importance because cardiac hypoxemia can lead to irreversible damage to the heart muscle known as myocardial infarction. In 2015, there were an estimated 7.29 million acute myocardial infarctions worldwide [6]. Myocardial ischemia is often indicated by chest pain. However, most of the affected individuals are unfamiliar or unaware of the typical symptoms. Consequently, they ignore the symptoms, resulting in a delayed diagnosis and treatment. Another reason for a delay in diagnosing myocardial ischemia is the availability of 12-lead ECG systems, which are limited to hospital settings or specialized health care facilities. Taken together, these circumstances often lead to a substantial delay between the onset of symptoms and the moment a medical doctor can make a correct diagnosis, thereby delaying appropriate treatment, which leads to a worse prognosis.

Apart from assisting in the diagnosis of myocardial ischemia, a 12-lead ECG can help accurately establish cardiac arrhythmias. Atrial fibrillation is the most common arrhythmia. Globally, there were an estimated 33.3 million prevalent cases of atrial fibrillation in 2015 [6]. Untreated atrial fibrillation causes up to 26% of all strokes [7], which can be prevented in 64% of patients with oral antithrombotic prophylactic therapy [8]. This shows that early diagnosis is of great importance.

Individuals with arrhythmias often have symptoms for a short period of time. These symptoms often subside when the affected individual visits the outpatient clinic, thus, severely reducing the sensitivity of a 12-lead ECG for establishing a diagnosis of transient ischemic heart disease. Therefore, the patients are often advised to wear a Holter monitor, which continuously records ECGs, for 24 or 48 hours. However, symptoms often do not occur during this relatively short monitoring period, which can result in a delayed diagnosis and treatment and can elicit feelings of insecurity within the affected individual. The abovementioned problems underscore the need to perform an ECG as soon as symptoms arise. This accelerates the diagnostic process, so that the patient can be treated faster, which will eventually limit the consequences of the disease.

### Existing Solutions and Their Limitations

With the rising number of CVD patients, the demand for home monitoring products that improve cardiac care is increasing. At present, two groups of ECG products are available for home monitoring.

The first group comprises products that are applied by health care professionals. This group includes Holter monitors that record very reliable information and are a key tool for diagnosing arrhythmias [9]. These systems are too complex to be used by laymen, and they are uncomfortable to wear. The sensor wires restrict the daily movements and interfere with the sleep of the wearer, and the electrode patches may damage the skin when removed. Therefore, from a user point of view, these systems are not ideal.

The second group comprises handheld ECG systems that are applied and used by patients themselves. These systems are, in contrast to the Holter systems, very easy to use and understand. Although these systems can, in some cases, support the diagnostic process of arrhythmias, they are not suitable for the detection and diagnosis of ischemia since the information is not as complete and reliable as the recordings of the standard 12-lead systems.

A short overview of the existing handheld ECG systems for consumer use follows. Note that well-being and fitness wearables, which measure heart rate, like the Fitbit, were not included in this overview since these are not classified as medical devices and are not suitable for diagnosing heart diseases [10,11].

Handheld ECG systems for consumer use generally consist of a measuring device that is controlled via an app on a mobile phone. The measuring equipment can be integrated into a patch [12] or a belt [13] for measurement on the chest. Alivecor developed Kardia, which, instead of using electrodes that are placed on the chest, requires the user to place his or her fingers onto two sensor pads, so that ECG data can be collected [14].

The aforementioned systems, having just a few leads, record too little information for diagnosing ischemia. The Smartheart Pro from SHL Telemedicine [15,16] is a system with multiple electrodes from which 12 leads are calculated. Electrodes are placed on a strap, which can be tied around the chest. However, synthesized ECGs from a reduced number of leads can approximate, but not duplicate, the tracings obtained using the standard leads [4]. This makes this system less reliable for ischemia detection compared with the standard 12-lead ECG systems.

In the past few years, research has been performed to integrate ECG sensors into textiles. Several 12-lead ECG shirts were developed for continuous health monitoring [17-19]. In these products, dry electrodes are integrated into the shirts. Unfortunately, dry electrodes are less robust and the signal-to-noise ratio is too low for diagnosing myocardial ischemia.

### Design Gap

To conclude, current ECG solutions for home monitoring are either easy to use but not reliable enough for diagnosing arrhythmias and ischemia or they are very reliable but too complex to be used by laymen. This shows that there is a need to design a novel 12-lead ECG device for home use that can be incorporated into daily medical protocols to speed up the diagnostic process. This new product-service system should enable patients themselves to make *reliable* 12-lead ECGs as soon as symptoms arise at home. By creating an *easy*-*to*-*use* product-service system, patients will be empowered to record essential data for diagnosing arrhythmias and ischemia.

This paper describes the development of a novel 12-lead ECG system for home use. The new system consists of a physical product, which includes the measurement system, and a service, which gives feedback during use. In this paper, we have focused on the embodiment of the physical product. The service is briefly explained. In addition, we have discussed the results of a recent technical performance study and a usability study that were conducted with a prototype of the new product to evaluate the design.

## Methods

### Development Process

**Figure 2:**
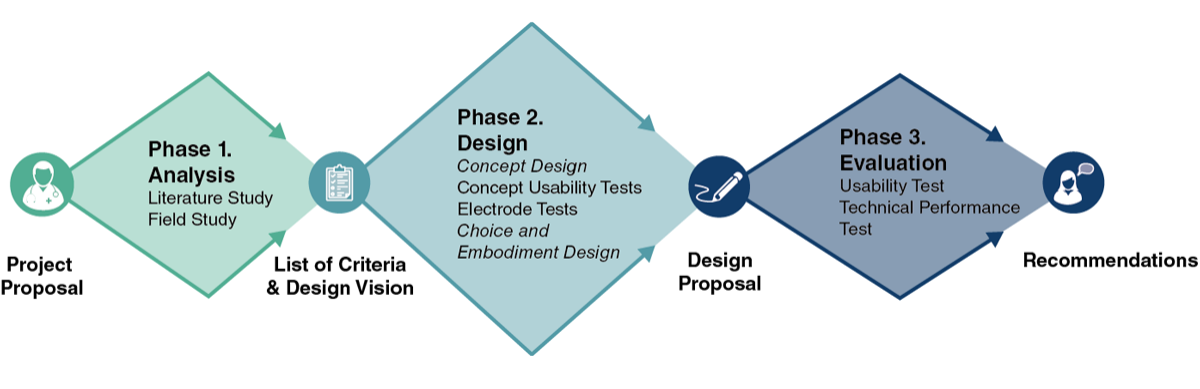
Visualization of the process.

During the design phase, the design methodology written by Roozenburg and Eekels [20] provided guidance to deal with all potential design problems in a systematic way. As can be seen in Figure 2, the process was split into 3 phases. We started the project with an analysis phase, resulting in a design vision and list of criteria. This phase was followed by a design phase where three concepts were developed and one of them was chosen. The chosen concept was developed further and designed in detail. In the third phase, a final prototype was created and several tests were performed to evaluate the product.

### Phase 1. Analysis: Design Vision and List of Criteria

We conducted a literature study and field research during the analysis phase of the project. We interviewed participants with arrhythmias or ischemia, cardiologists, and specialized nurses and observed standard ECG procedures. Then, we mapped out the insights from the literature study and the field research and defined a list of design criteria and a design vision.

### Phase 2. Design: Process and Prototypes

The two core values that were highlighted in the design vision are *reliability* and *ease of use*. These values served as a guide through the design process. For each core value, the most important tests and subsequent decisions are mentioned in the next paragraphs. Note that, in reality, the mentioned activities formed an iterative process together with the design steps. The design vision served as a start of the design process. More than 60 ideas were generated, and the most promising ideas were developed into three concepts [20]. Prototypes of these concepts were made, which were evaluated with users on usability and comfort. Parallel to this, we performed technical performance tests with different electrode-skin contact designs. We further developed the best scoring concept into a final design proposal.

#### Designing an Easy-To-Use Electrocardiographic System

During the whole process, we used a user-centered design approach [21]. Potential users of the ECG system were involved throughout the process to ensure that the product fits the needs and expectations of the user group. In order to evaluate the comfort and usability of the different concepts, we made simple mock-ups of the three concepts. We asked 5 participants to use the mock-ups and assess them on device comfort and usability in a structured interview. All participants gave informed consent before participating.

As shown by the empirical studies discussed by Kanis [22], this user trialing can be seen as an obvious way to enable designers to accommodate prospective user activities in everyday product design.

The participants were on average 65.6 (SD 16.2) years old. Of them, 3 were women and 4 participants had experienced an ECG test before. None of the participants had a heart disease.

First, the project goal and the procedure were explained to the participants. Second, each participant was asked to try to put on each mock-up with the help of simple visualizations that show how the device is worn. We measured the time of the actions and observed the actions. After the use of the mock-ups, the participants were interviewed.

#### Designing a Reliable Electrocardiographic System

To design a feasible solution for laymen to record reliable ECGs at home, we took several main steps. First, correct placement of the electrodes needed to be facilitated for people with different shapes and sizes. To identify the difference in the distance between the electrodes, we retrieved the three-dimensional (3D) body scan data of approximately 1250 people from the Delft University of Technology database WEAR [23] and calculated P5 (5th percentile) and P95 (95th percentile) of the circumference under the bust (for the Dutch population, only measured for females). The electrodes were positioned at a P5 and P95 3D scan following the anatomic guidelines of electrode placement of a standard 12-lead system. After this, the difference was calculated.

Second, optimal skin-electrode contact is needed to ensure that the signal can be captured without recording too much noise. The aim of the electrode-skin contact tests was to find out which concepts ensured the best skin-electrode contact on the chest.

We gathered several electrode samples that enhanced electrode-skin contact. 3M Red Dot Electrodes [24], two types of reusable electrodes of PLUX Wireless Biosignals [25], and electrodes with foam from Somnomedics [26] were used. To compare the different electrodes, we made an ECG with a three-electrode bipolar lead system [27]. To check how well the electrodes were connected to the skin, we measured the electrode impedance and interpreted the ECG recordings. By comparing the ECG results, we identified the best performing electrode.

### Phase 3. Evaluation: Usability Study and Technical Performance Study

Based on the different test results, we selected one concept for further development in detail, which resulted in a final design proposal. To evaluate the proposal, we created a prototype for the final usability study and technical performance study.

#### Usability Study

We performed the final user tests to evaluate the user-friendliness of the newly designed device and to identify the bottlenecks in its use. Six participants from the potential target group were invited to use and assess the prototype. All participants were older than 60 years with a mean age of 73.7 (SD 7.8) years. The user group included 4 females and 2 males. Five participants had experienced an ECG test in the past, but no one had seen the new design before. All participants gave informed consent.

First, the project goal and the procedure were explained to the participants. Second, a preliminary version of the manual was presented to each participant on a laptop. We asked each participant to try to put on the prototype with the help of the manual while the researchers timed and observed the users’ actions. After the use of the device, the participants completed structured interviews. We asked questions about the perceived usefulness and ease of use since these factors greatly influence the users’ decision about how and when to use the new product [28].

#### Technical Performance Study

Following the usability test, a first technical performance test was performed. In this test, we checked whether all the electrodes in the final prototype made good contact with the skin of one female participant. We also checked whether the belt fitted well and investigated which parameters needed to be adjusted.

At the outpatient clinic in the hospital in Zaandam (Zaans Medisch Centrum), the wires of a standard 12-lead resting ECG monitor were connected to the electrodes on the prototype of the belt. A series of ECGs using the standard equipment of the 12-lead resting ECG system were made first as a reference. Subsequently, a 12-lead ECG was made with one prototype 6 times. After each recording, the belt and straps were taken off and the electrodes were replaced. The test was performed multiple times to check whether the results were reproducible.

## Results

### Phase 1. Analysis: Design Vision and List of Criteria

Based on the literature study and field research, design criteria were formulated. The most important design criteria are described below:

1. The product-service system should enable a patient without experience in electrocardiography to make an ECG of medical quality himself or herself.
2. The product should contain 10 electrodes, from which 12 leads can be derived.
3. The product-service system should contain a communication channel for quick interpretation of the ECGs by a health care professional.
4. The location of the precordial electrodes should never differ more than 3 cm from the anatomically defined locations in the body [29].
5. The product should be put on and used by the person whose ECG needs to be recorded.
6. ECGs should be recorded with the patient in a supine position.
7. Users should be able to make an ECG within 10 minutes. Somerville et al reported that the average time to perform a conventional 12-lead ECG is 10.6 minutes [30].
8. The patient should be able to communicate his or her complaints together with the recordings to the health care specialist in a simple way.
9. The results of the test should be clearly communicated to the patient.

The design vision was stated as follows: “We want to design a product-service system that enables subjects to make a reliable 12-lead ECG at the moment they have complaints at home. With the help of an easy-to-use product-service system, patients are empowered to record essential data for diagnosing arrhythmias and ischemia.” Two core values can be highlighted in this vision: reliability and ease of use. The product should enable the user to make 12-lead ECGs of good quality, suitable for interpretation by a cardiologist; at the same time, the product-service system should be very easy to use and understand.

### Phase 2. Design: Process and Prototypes

**Figure 3:**
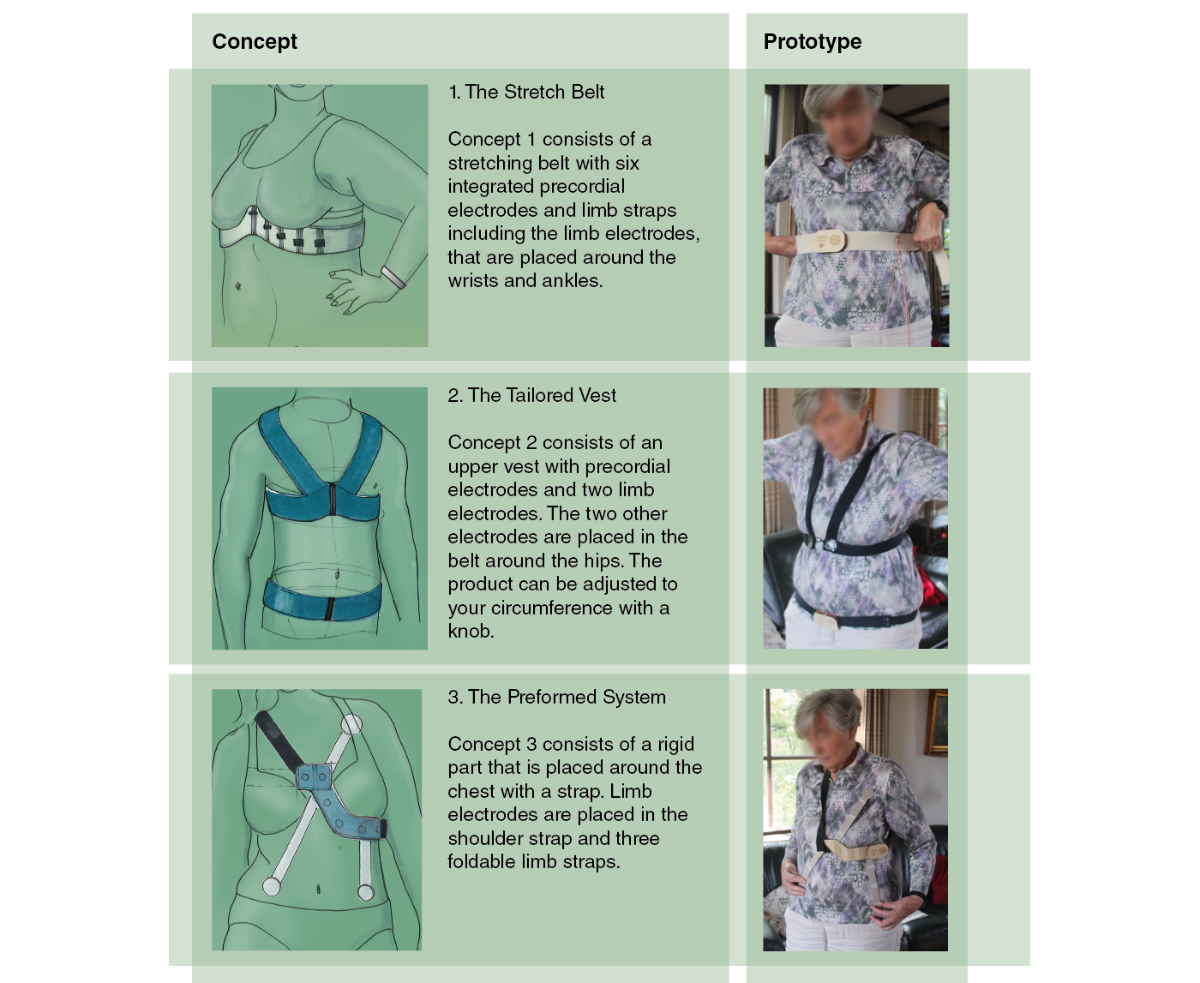
Concept design and mock-ups.

In this section, the preliminary design concepts are presented first, followed by an explanation of the final design. Figure 3 shows the sketches of the three design concepts and the three mock-ups that were used in the concept user tests. Concept 1, from now on called *ECGraph* was chosen for further development. This system consists of a belt and 4 limb straps. When placed around the chest, the belt will stretch and the electrodes will move with respect to each other. In this way, the electrodes will automatically be placed at the right location on the body and would not need to be placed at the right location one by one, like in the conventional systems. Positioning the middle part of the product in the middle of the body and above the nipples is sufficient. This makes the product very user-friendly. Note that the limb electrodes are placed on the actual limbs like in the current hospital procedures, which increases the acceptance of the product by cardiologists.

**Figure 4:**
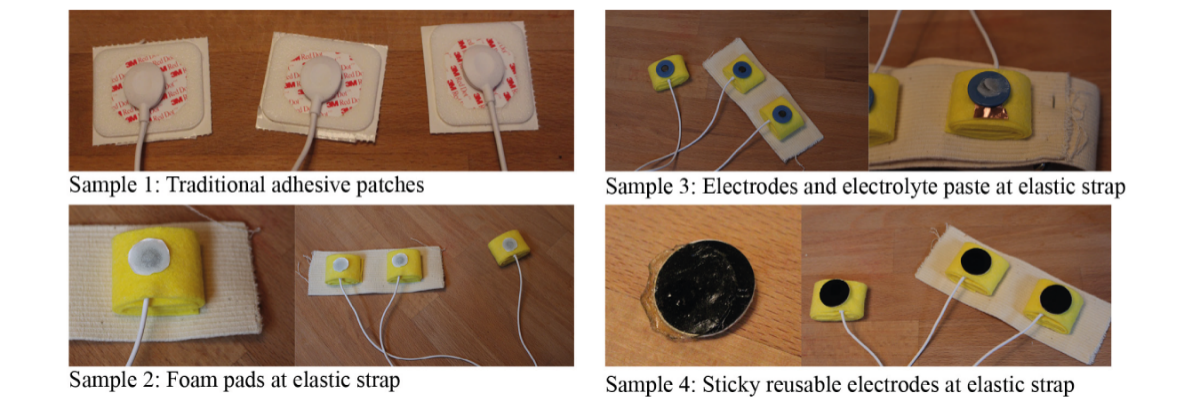
Samples used in electrode-skin contact tests.

We chose the design of the electrodes with the help of electrode-skin contact tests. The samples that were tested are shown in Figure 4. The test results showed that the Ag/AgCl-gelled disposable electrodes (samples 1, 2) made the best contact with the skin. If users have to add the gel manually, like for sample 3, good contact cannot be guaranteed.

#### Final Design

**Figure 5:**
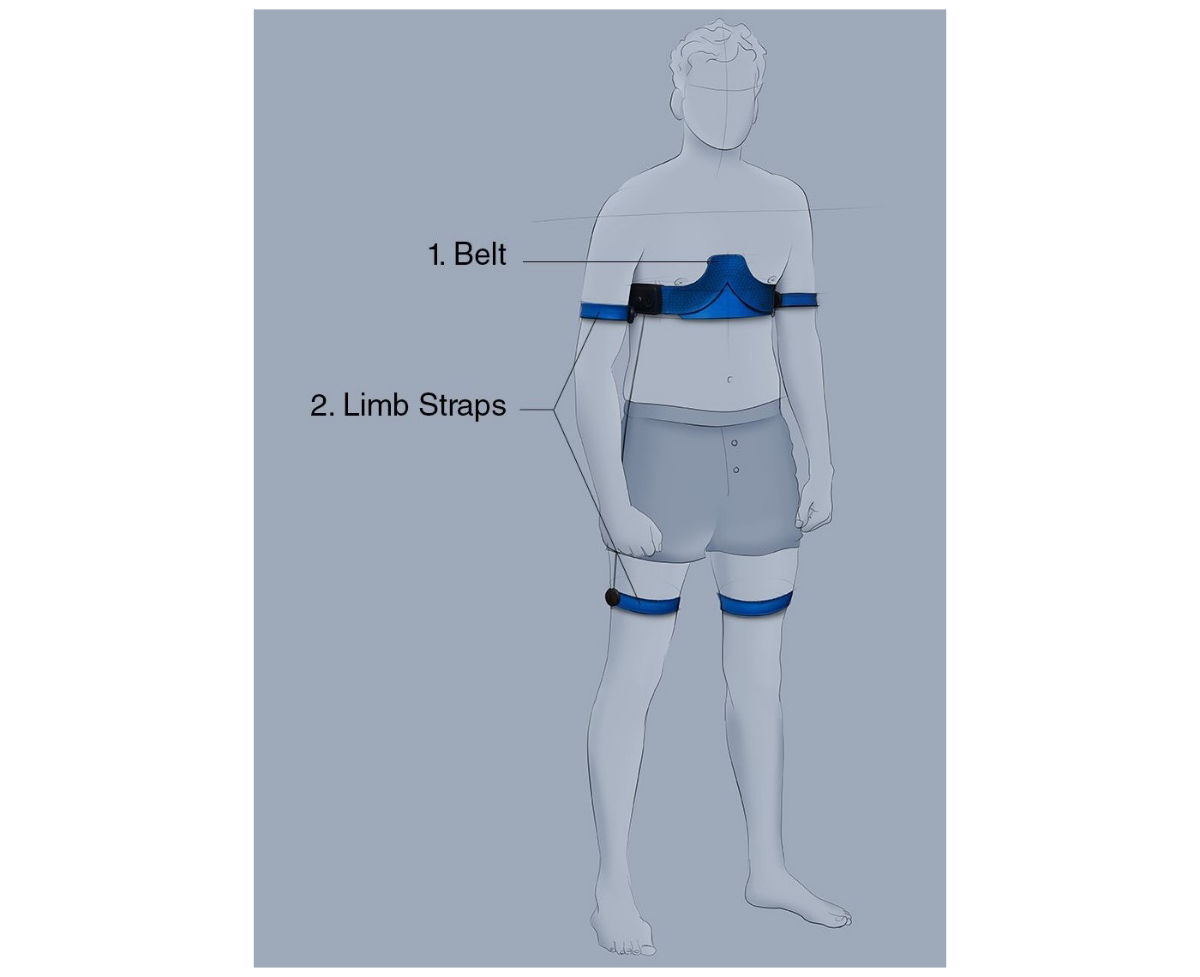
Positioning of the final design.

**Figure 6:**
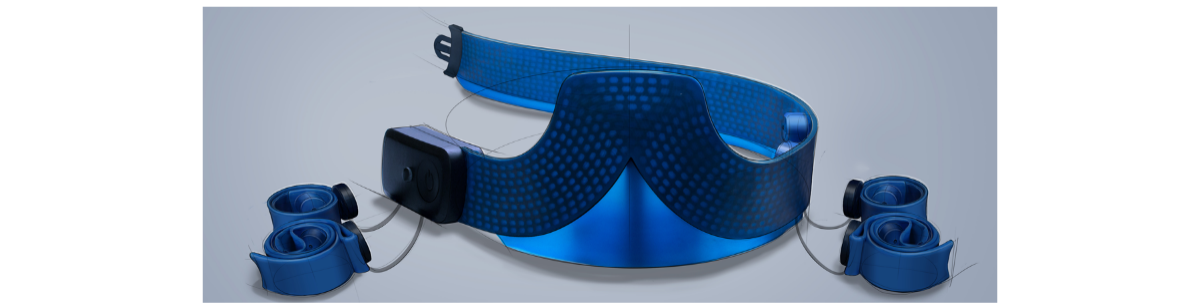
Final design: main belt connected with the limb straps through rolling mechanisms.

Figures 5 and 6 illustrate the final design. *ECGraph* is a stretching belt (see #1 in Figure 5) that is placed under the bust. The belt is connected to 4 straps (see #2 in Figure 5) that need to be placed around the limbs and contain the limb electrodes. The 6 electrodes of the precordial leads are integrated into the belt, and with a simple closure system, the belt can be placed around the chest.

The limb electrodes are integrated into the 4 straps. The straps are preformed so that they can be wrapped around the limb with one hand. They are connected to the belt through rolling mechanisms to ensure that the wires do not get entangled. The limb straps need to be connected to the belt through wires because all the electrodes and the associated readout circuit need to have a common ground. In case a wireless connection without a ground cable is used, the ECG measurement will be significantly deteriorated due to noise and interference (Dr J Xu, personal communication, December 8, 2016).

**Figure 7:**
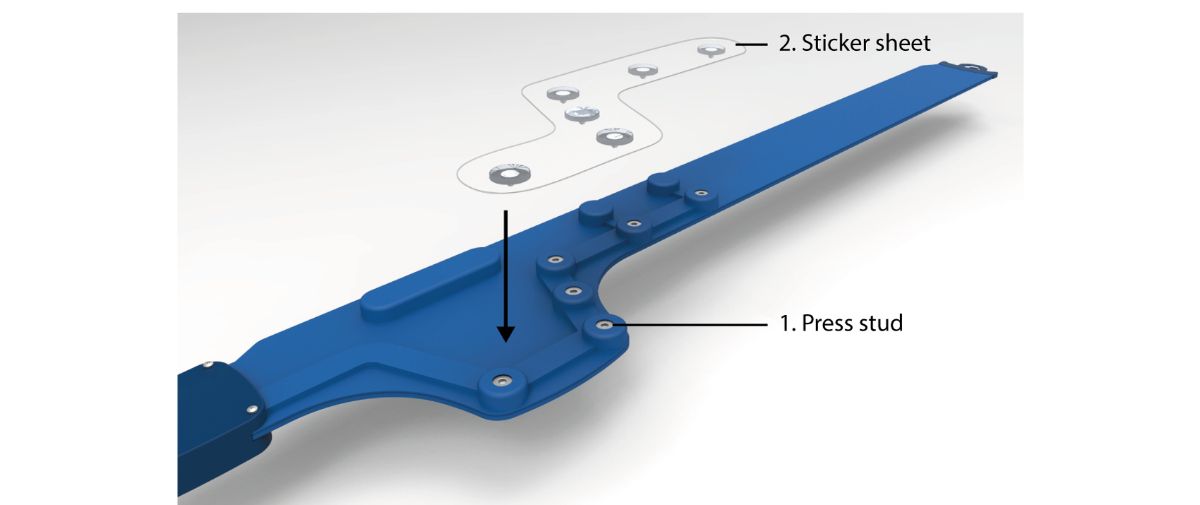
The electrodes are preplaced on a sticker sheet and connected to the belt using press studs. When the electrodes are connected, the sheet can be pulled off.

Standard disposable Ag/AgCl electrodes (sample 1) are integrated into the final design because they ensure good electrode-skin contact. The adhesive part of these electrodes is removed because the belt will keep the electrodes in place during the recording, enhancing comfort. Figure 7 shows how the electrodes are placed on the belt using a commonly used press stud system. For ease of use, the precordial electrodes are already positioned on one sticker sheet that can be easily pulled off.

**Figure 8:**
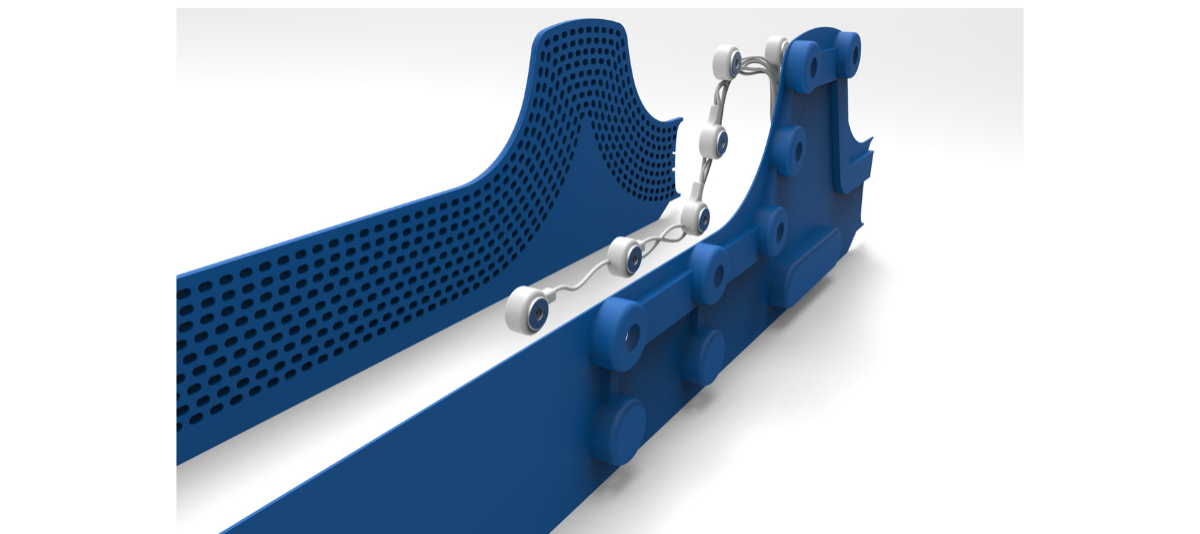
The electrode system is sandwiched between two layers of cast rubber, one of which is perforated to create a larger strain.

**Figure 9:**
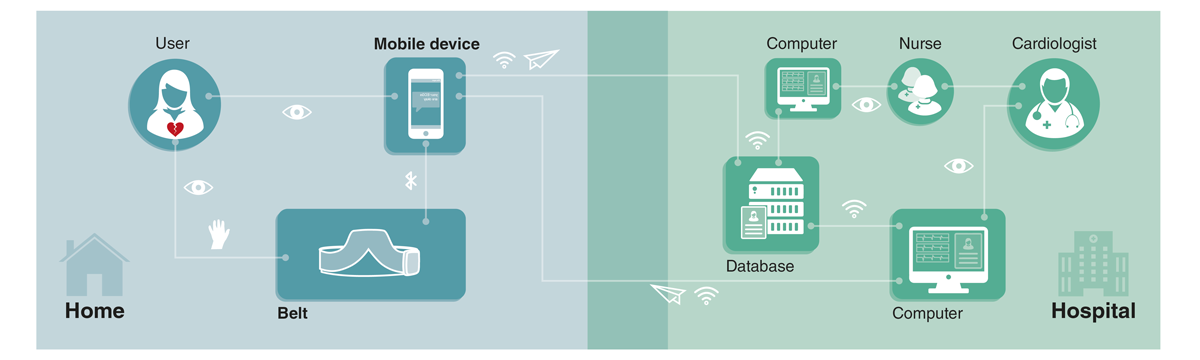
Overview of the product-service system.

Figure 8 shows how the electrode system is integrated into the belt. The belt will be available in two sizes to minimize pressure on the body for larger individuals, once again enhancing comfort. Figure 9 presents an overview of the product-service system. The user can record the ECGs by putting on the belt and the straps. The user’s phone can connect to the belt via Bluetooth. Once the Bluetooth connection is made and the user turns on the device, the system automatically checks whether the electrodes have made good contact with the skin. Afterwards, the user can record the ECGs.

**Figure 10:**
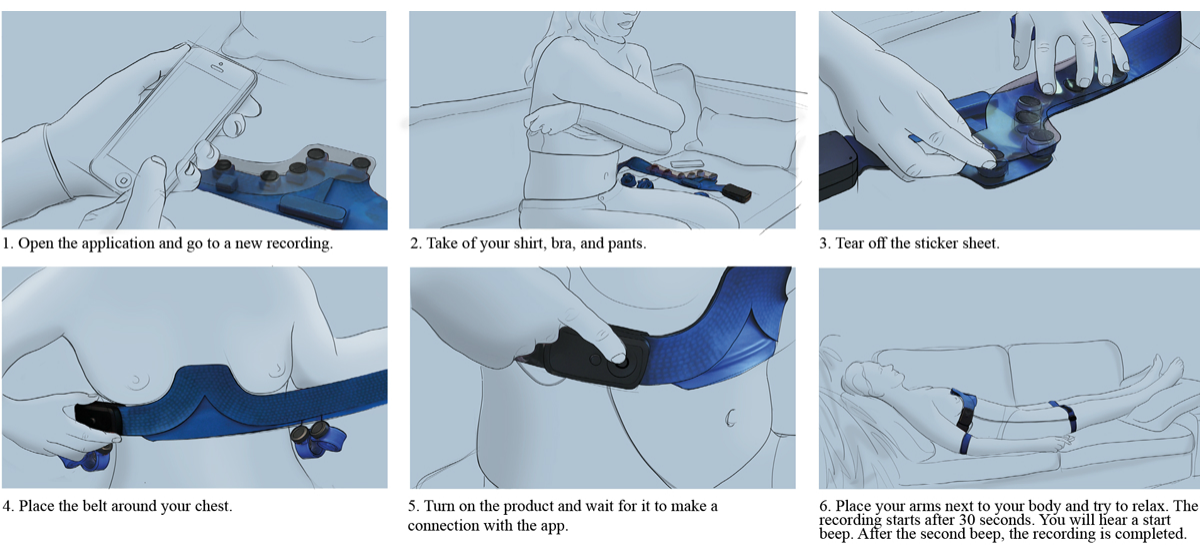
A selection of steps from the manual.

The user can store the ECGs and send them, together with a description of his or her complaints, to the hospital database via Wi-Fi or a 4G network. The nurses or medical assistants examine the ECGs. When excessive noise is detected by the nurses and when the ECGs are uninterpretable, the patient is asked to repeat the process. When the ECGs are of good quality, they are electronically forwarded to the cardiologist. The cardiologist interprets the ECGs and formulates an advice based on the results. The advice is sent to the patient in a text message via the app. It lets the patient know whether direct action is required. To envision the service, a more detailed service blueprint was made and some screens of the app were created (see Multimedia Appendix 1) [3]. To make the user feel confident about recording the ECGs and to ensure that he or she can record ECGs of good quality, the user is asked to record a “test ECG” at the moment he or she receives the product. After opening the app, the user first makes a connection with his cardiologist. After the cardiologist confirms the connection, the user is asked to record a test ECG with the help of the app. The ECGs are checked by the cardiologist, and the user gets feedback on the results. Last, during product use, the user is guided by a visual manual that is shown in the app. Some important steps from this manual are shown in Figure 10. In this manual, all the important requirements of use are highlighted.

### Phase 3. Evaluation: Usability Study and Technical Performance Study

In this section, the results of the final usability study and technical performance study are presented. We used these results to evaluate the final design.

#### Usability Study

The user tests proceeded smoothly. All users were able to put on the prototype within 8 minutes. Table 1 shows the main issues that were identified during the usability study (n=6).

All participants placed the limb straps at the correct locations during the test. Concerning the placement of the belt, one participant had the tendency to place the belt on the chest at a level lower than that in the picture (Issue 1). All participants had the tendency to ask questions about whether they were using the prototype in the right way. They did not feel confident during handling the device, although they were actually doing it very well. Besides, they explained in the interviews that they would feel even less confident using the device when they were experiencing symptoms of a heart disease. On the other hand, all participants assented that they would feel more confident if they had used the product before.

#### Technical Performance Study

**Figure 11:**
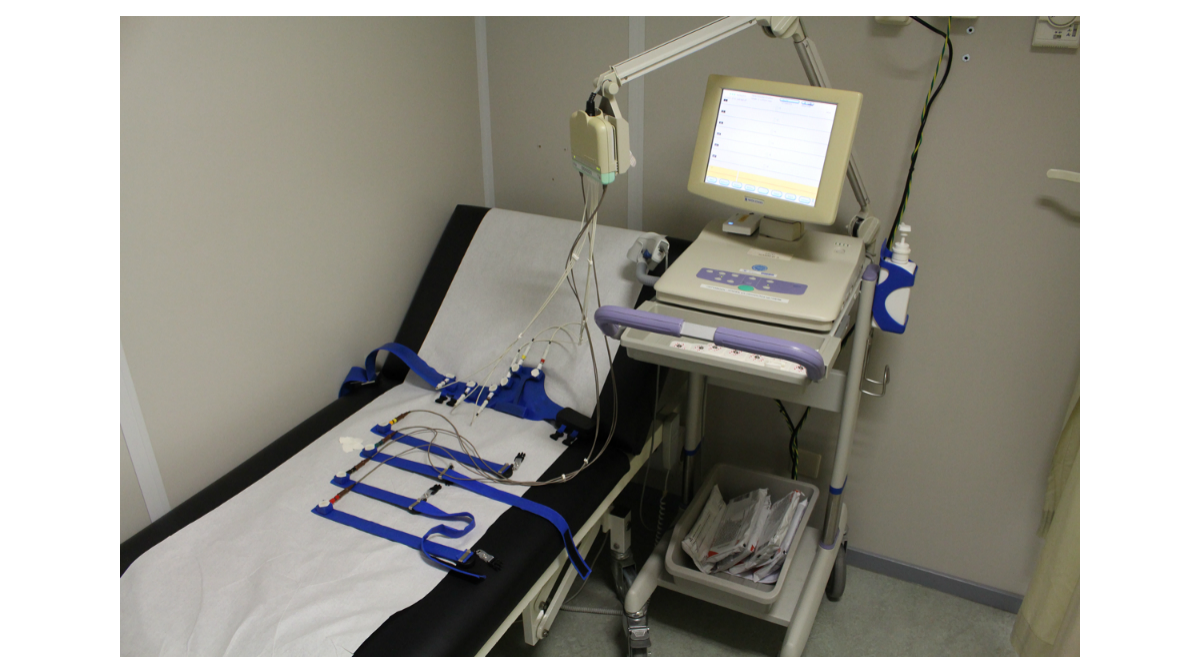
The standard 12-lead monitoring equipment connected to the prototype.

**Figure 12:**
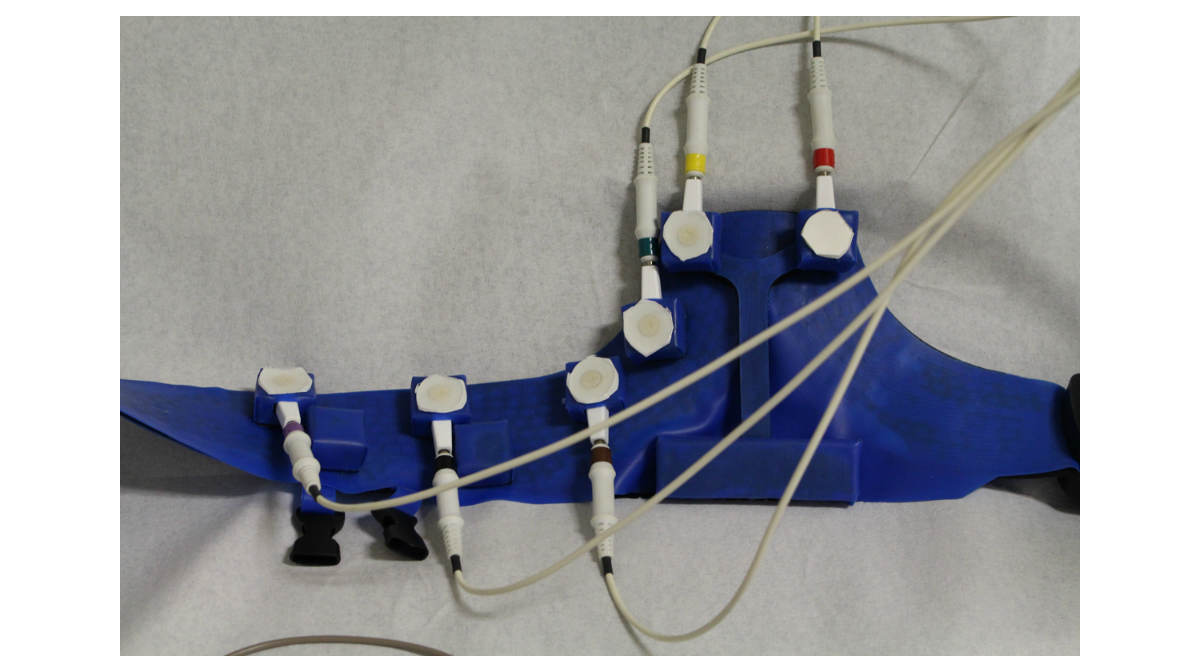
Detailed picture of the prototype (blue) connected to the standard electrocardiogram monitoring equipment. Note that in the final design, the electrode wires will be integrated into the belt.

**Figure 13:**
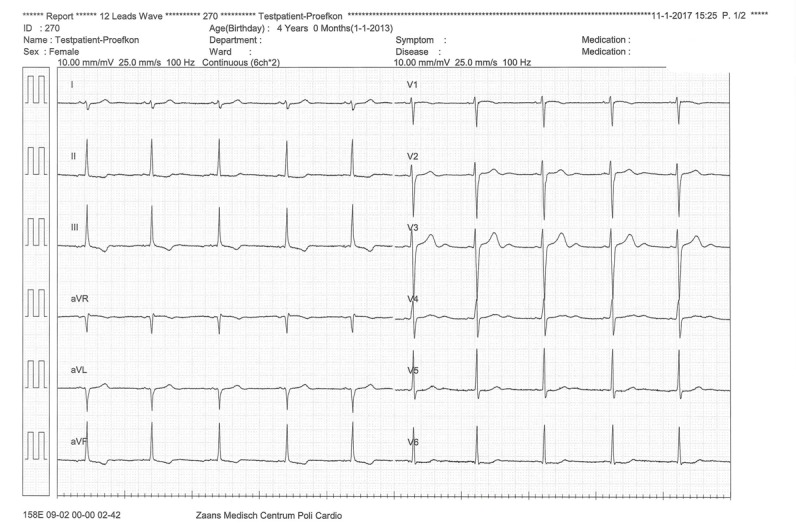
Standard electrocardiogram recording made using adhesive patches. Limb electrodes were placed on the limbs.

**Figure 14:**
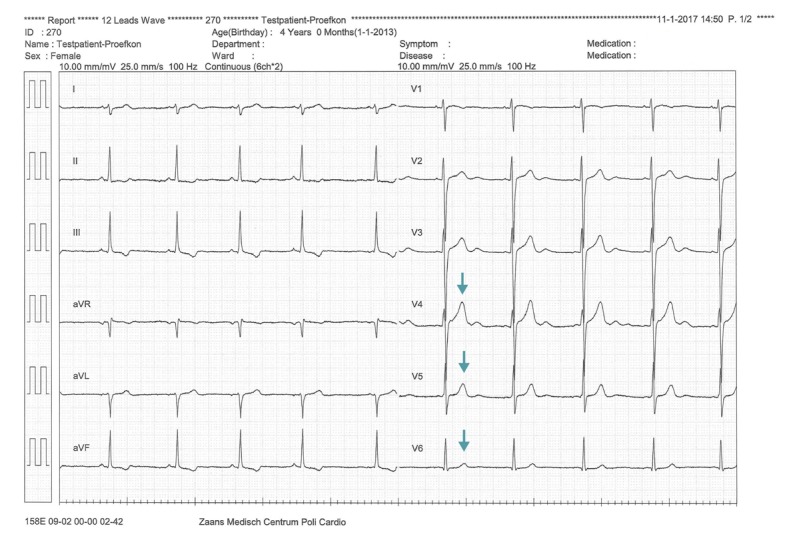
Electrocardiogram recording made using the prototype.

Figures 11 and 12 show the set-up during the technical performance test. A series of ECGs using the standard equipment of the 12-lead resting ECG system, were made first as a reference. Subsequently, a 12-lead ECG was made using the prototype 6 times. Figure 13 depicts a standard recording, and Figure 14 shows an ECG recording made using the prototype. The ECG results were compared by a cardiologist, who confirmed a good reproducibility of the ECGs made with the prototype.

Second, the standard ECG was compared with the ECGs made with the prototype. It was noticed from the graphs that the placements of V4 till V6 were slightly different when the prototype was used. As indicated by the arrows in Figure 14, the T-waves of V4 till V6 were larger than those in Figure 13.

This can be explained by the fact that in the test with the standard ECG system, the patches were placed a little lower because the participant was wearing a bra. Although the signal-to-noise ratio appeared to be lower when the prototype was used, this did not influence the diagnostic characteristics of the ECG.

**Table 1:** Major issues identified during the usability study.

Issue #	Participants	Task	Issue
1	P4	Placement of the belt	The belt was placed too low around the chest.
2	P1, P2, P5, P6	Placement of the limb straps	Although every participant placed the straps around the right limb, the majority of them were not sure about which strap should be placed around which limb.
3	P3, P6	Placement of the limb straps	Some participants were not sure about the location of the electrode on each limb. They were not informed that this was not important for measurement reliability as long as the electrode was placed on the correct limb.
4	P2, P4, P6	Check before recording	Several participants were not sure whether they will feel confident enough to use the system when they have real complaints.

## Discussion

### Principal Findings

In this paper, we described the design and development of *ECGraph*, which is the first product-service system that enables individuals to record reliable standard 12-lead ECGs at home at the moment they have complaints. The design vision contained two core values. First, the novel 12-lead ECG system must record ECGs of clinical quality. The measurements must be reliable and suitable for clinical interpretation. Second, individuals using the system need to be able to record the ECGs themselves. Therefore, an easy-to-use product-service system had to be made. We have discussed whether the designed product-service system (*ECGraph*) adheres to these core values. Recommendations to improve these key aspects are presented as well.

### Technical Performance

Several design aspects contribute to the reliability of the system. First, the new ECG system makes use of 10 electrodes, just like the standard 12-lead ECG systems. In contrast to many competitors who use measurement systems with fewer leads, we designed a full 12-lead system, which has been shown to be more reliable [4]. Disposable wet electrodes, which ensure good skin contact, are placed in the belt and straps. These features offer an advantage over the existing products for home use, namely that the product can be used in clinical practice for diagnosing infarctions and arrhythmias.

A first technical performance test with the final prototype was performed to find out whether the new design allowed the electrodes to make a good contact with the skin. From this test, we concluded that ECGs of clinical quality could be made. Five repetitive ECG measurements confirmed the system’s good reproducibility.

### Usability

In the design phase, ease of use was prioritized. Several user tests were performed to evaluate the ease of use and to improve the design. This led to a lot of design elements that contributed to the ease of use of the device. The precordial electrodes were integrated into a belt; the replacement of the patches was facilitated by a sticker sheet, which contains the precordial electrodes; preformed arm straps were made; and rolling mechanisms for the wires were used. Besides, the product has very simple interface buttons, and an app supports the user during product use.

In the final usability test, all the users were able to put on the product correctly within 8 minutes. We expect that the product can be used much faster once the users familiarize themselves with it. One participant placed the belt slightly lower on the chest than pictured in the manual. This would influence the ECG results, but it can be solved by adding an extra remark to the digital manual in the app stating that the top electrodes need to be placed between the breasts, above the nipples.

From the final usability test, we discovered that lack of confidence among the users during the procedure was a major issue. To make the users feel more confident, the manual should be improved, and it should contain some extra remarks and check points. We found that the participants wanted more information about whether they were handling the product correctly. This emphasizes the importance of making a “test ECG” that will be checked by the cardiologist when a user receives the product for the first time. An instruction video or demonstration in the hospital can also increase confidence during use.

Last, some design elements led to ease of use for the health care professional. The use of a traditional, 12-lead system facilitates implementation in the current health care system. On top of that, the effort required from the cardiologist is minimal since he or she only has to send a message with the outcome of the test to the user.

### Limitations

Because of the short time span of this project, there are several limitations that warrant discussion. First, during the usability test, the participants were wearing clothes. Thus, the available wet electrodes were not placed in the prototype during the test. In the next phase, a new user test needs to be performed where the participants will have to take off their clothes and the wet electrodes will be placed in the prototype to simulate the real user scenario.

Second, the prototype was made from laser-cut segments of polychloroprene and latex because there was insufficient time to create a mold and fabricate a belt by casting. These segments were glued together, which made the belt less robust than the intended product. The fragility of the prototype may have influenced the perception of the product and, therefore, the results of the user tests.

Third, during the technical performance test, ECGs of a person with a small thoracic circumference (700 mm) were made. We currently have no data on the quality of ECGs in persons with a relatively large thoracic circumference; this should also be tested in the future.

Last, in this paper, we described the first step, namely the design phase, of creating a 12-lead ECG system for home use that can be incorporated into clinical practice. The system has not yet been put into test on patients who are suffering from a cardiac disease. A clinical study to test whether the new device can speed up the diagnostic process of patients with a cardiac pathology has been planned as the next step.

### Conclusions

In this project, a new 12-lead ECG system for home use was developed. The product (*ECGraph*) is designed to be more user-friendly than current hospital ECG systems, and the product architecture enables integration of ECG components that have a superior sensitivity for diagnosing cardiac pathology compared with outpatient surrogate ECG devices. By performing a thorough context and user research, we have tried to create an accessible product for both patients and health care professionals. We believe that *ECGraph* has great potential to be incorporated into current clinical practice models and is a step forward toward faster diagnosis and treatment of patients with cardiac pathology.

## Abbreviations

CVD: cardiovascular diseases
ECG: electrocardiogram/electrocardiography

## Appendix

Multimedia Appendix 1 App screenshots.
