# Supplementary figures and images for: A Novel 12-Lead Electrocardiographic System for Home Use: Development and Usability Testing
Source: JMIR Mhealth Uhealth. 2018 Jul 30;6(7):e10126. doi: 10.2196/10126 (PMC6090173; doi:10.2196/10126)

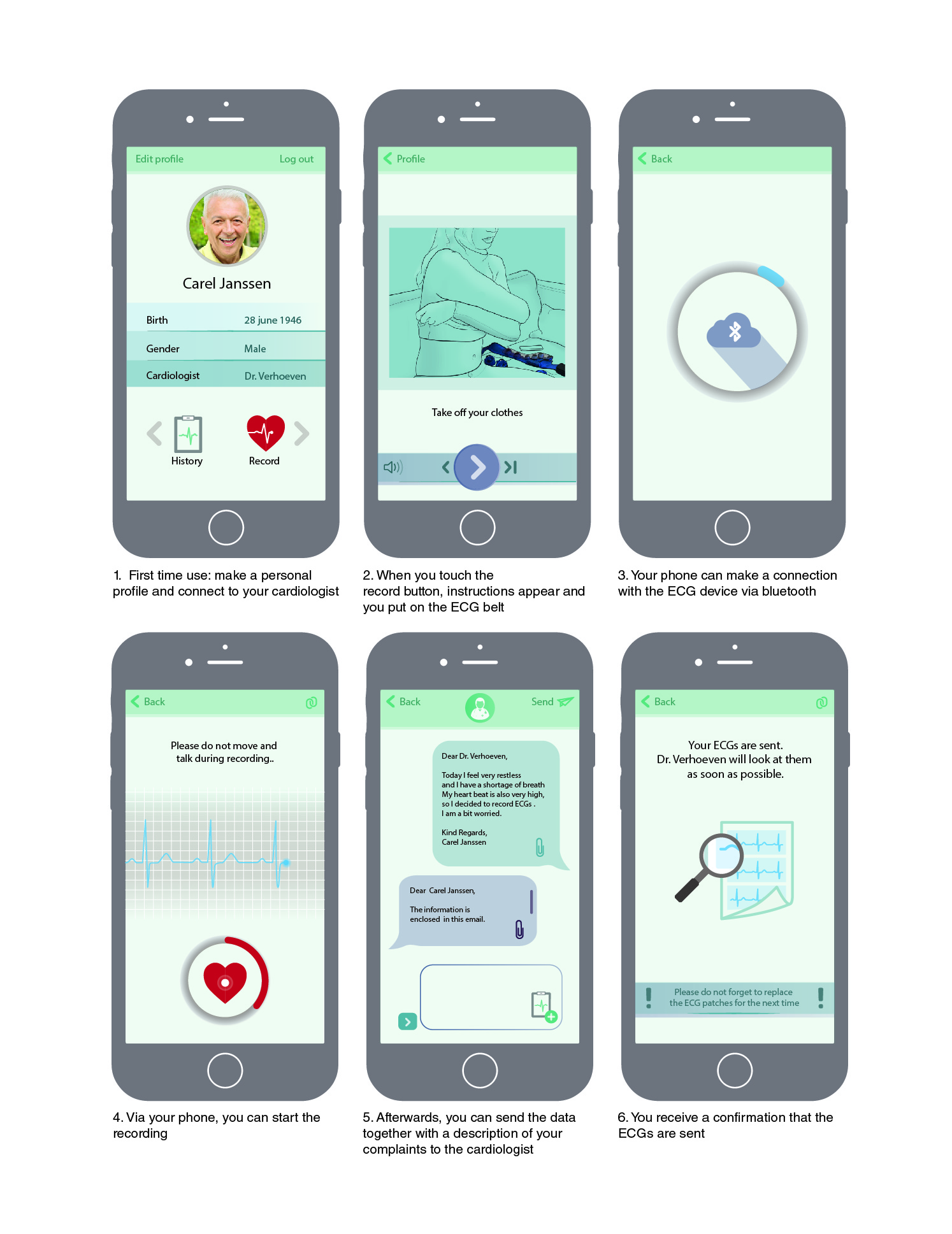

Supplement: Multimedia Appendix 1 [file mhealth_v6i7e10126_app1.jpg]
